# Hypothermia as a predictor for mortality in trauma patients

**DOI:** 10.1186/cc14387

**Published:** 2015-03-16

**Authors:** K Balvers, JM Binnekade, C Boer, JC Goslings, NP Juffermans

**Affiliations:** 1 Academic Medical Center, Amsterdam, the Netherlands; 2 VU University Medical Center, Amsterdam, the Netherlands

## Introduction

Previous studies reported hypothermia as an independent predictor for mortality. However, different cutoff points were used in these studies and external validation has never been applied. The aim of this study was to quantify the net effect of hypothermia on admission to the ICU on the 28-day mortality and to test the predictors from the developed model in another level 1 trauma center with a comparable patient population to validate the model.

## Methods

A retrospective cohort study was performed in adult trauma patients admitted to a level 1 trauma center and who were transferred to the ICU between 2007 and 2012. Different cutoff points for hypothermia were compared to find the best definition for hypothermia. Logistic regression analysis was performed to quantify the net effect of hypothermia on admission to the ICU on 28-day mortality and to develop a model with predictors. The developed model was externally validated in data from another level 1 trauma center with a comparable patient population.

## Results

In total, 722 trauma patients were included, of which 300 patients were hypothermic. The mortality in the hypothermia group was significantly higher than in normotherm patients (OR = 3.73, 95% CI = 2.02 to 7.13, *P *< 0.001). A cutoff point of 36°C was observed as the best threshold for hypothermia (sensitivity 74%, specificity 56%). Besides hypothermia, other predictors found for 28-day mortality were APACHE II score corrected for temperature, minimum thrombocytes in first 24 hours and urea and included in the final model with an AUC of 0.89 (95% CI = 0.85 to 0.92). External validation of the model was associated with a predicted probability of an AUC of 0.64 (95% CI = 0.51 to 0.77).

## Conclusion

Hypothermia, defined as <36°C, is associated with an increased 28-day mortality. The discriminative ability of the developed model for predicting mortality in a new patient population is moderate.

